# Digital System Design for Quantum Error Correction Codes

**DOI:** 10.1155/2021/1101911

**Published:** 2021-12-15

**Authors:** Othman O. Khalifa, Nur Amirah bt Sharif, Rashid A Saeed, S. Abdel-Khalek, Abdulaziz N. Alharbi, Ali A. Alkathiri

**Affiliations:** ^1^Electrical and Computer Engineering Department, International Islamic University, Gombak, Malaysia; ^2^Department of Computer Engineering, College of Computers and Information System, Taif University, P.O. Box 11099, Taif 21944, Saudi Arabia; ^3^Department of Mathematics and Statistics, College of Science, Taif University, P.O. Box 11099, Taif 21944, Saudi Arabia; ^4^Department of Physics, College of Science, Taif University, P.O. Box 11099, Taif 21944, Saudi Arabia

## Abstract

Quantum computing is a computer development technology that uses quantum mechanics to perform the operations of data and information. It is an advanced technology, yet the quantum channel is used to transmit the quantum information which is sensitive to the environment interaction. Quantum error correction is a hybrid between quantum mechanics and the classical theory of error-correcting codes that are concerned with the fundamental problem of communication, and/or information storage, in the presence of noise. The interruption made by the interaction makes transmission error during the quantum channel qubit. Hence, a quantum error correction code is needed to protect the qubit from errors that can be caused by decoherence and other quantum noise. In this paper, the digital system design of the quantum error correction code is discussed. Three designs used qubit codes, and nine-qubit codes were explained. The systems were designed and configured for encoding and decoding nine-qubit error correction codes. For comparison, a modified circuit is also designed by adding Hadamard gates.

## 1. Introduction

Quantum computing is a development of computer technology that is based on the theory of quantum which is quantum mechanics. Quantum computers can be used to solve problems with high difficulties which cannot be solved by normal computers [1–3]. One of the fundamental principles of quantum mechanics is that it is possible to create superpositions of different physical configurations. A simple example of this principle is a hydrogen molecule with only one electron. An electron in the molecule could belong to the left proton or the right proton. Because it is a quantum object, it can belong to both. It is in a quantum superposition of being left and right.

The idea of quantum error correction (QEC) was driven by the explosion of interest in quantum computers. A handful of people, starting in the 1980s, began to ask if a computer operating according to the laws of quantum mechanics might be more powerful than ordinary classical computers, which obey classical laws. QEC is considered the backbone of quantum information processing since it converts an unmanageable continuum of errors into a manageable discrete set. The theory of error correction was developed to correct classical coding for noisy channels [4–7].

In a normal computer, the information is encoded into bits and being transferred as either 0 or 1. Meanwhile, in quantum computing, the information is stored into quantum bits or qubits and being transferred as either 0, 1, or in a superposition state which is 0 and 1 at the same time. In general, one qubit can be represented as *α*|0) + *β*|1) in the Hilbert space and can be used to carry the information [2]. In the quantum case, the superposition of the 2*n* possible states must be specified. For example, if there are 300 qubits, it would need 2300. It is a very big number of particles and gives a sense of enormous complexity that can be found in the quantum area. Hence, the purpose of quantum computing is to control the complexity by performing certain calculations much faster than any normal computer ever could.

A quantum channel is a channel that transmits quantum information in quantum bits or qubits. Quantum error correction, on the contrary, protects a qubit of information from errors that might be caused by decoherence and other quantum noise in quantum computation and communication. The first quantum error-correcting code was proposed by Peter Williston Shor. Shor has proposed a method of using the entanglement of nine qubits' code to keep the information to correct the quantum error [3]. After that, a wider class of quantum error corrections has been discovered.

In classical computation, the corrupted information can be corrected by a redundancy scheme. It is the simplest way of error correcting in which firstly, it will copy the information to be preserved. Later, when there is a noisy error in the information, it will be corrected simply by taking a majority. However, in quantum states, it is impossible to use the redundancy scheme due to the “no-cloning” theorem. This paper will discuss a new system design for the quantum error correction code which is nine-qubit codes.

The remainder of this paper is organized as follows: Section 2 discusses the related works of the quantum error corrections.  Section 3 presents the proposed quantum error correction code.  Section 4 discusses the digital system design (DSD) for the quantum error correction code. The paper is concluded in Section 5, where conclusion remarks and recommendations for future designs are stated.

## 2. Related Work

There are many methods used to correct the error of quantum computing where many research on quantum error-correcting codes. Wada et al. in [4] proposed the construction of quantum error-correcting codes by designing the circulant permutation matrix which is obtained from the parity check matrices. A sufficient condition of the parity check matrices has been derived so that the entanglement-assisted quantum error-correcting code (EAQEC) only needs one maximally entangled quantum state. In addition, this paper also presented that proposed parity check matrices have no 4-cycle to show high error-correcting performance in the sum-product decoding.

Niwa et al. in [5] constructed a simulation to investigate the effect of the quantum error-correcting code in five-qubit codes, seven-qubit codes, and nine-qubit codes and their fault tolerance operations when the error correction process produces an error. This study showed that the seven-qubit code scheme is the most effective in [4–10] or less of decoherent rate and when the standard operational error is 10–3 or less. In addition, the error correction process in the seven-qubit code is operated at every 50 to 200 main gates even though there is that both errors exist.

The study conducted by Ahn et al. in [6] purposed the usage of quantum feedback control for correcting the quantum errors which are produced by weak continuous measurement. The explicit scheme is derived using stabilizer formalism which includes feedback and an additional constant Hamiltonian to protect the code space when the time and location of spontaneous emission are known and correcting unitary is applied instantaneously. This paper shows an insight into the detected-spontaneous emission error correction with driving Hamiltonian that can decrease the amount of the required redundancy. The quantum feedback provides a way to analyze the continuous feedback mechanisms by considering the unraveling of the master equation.

Yin et al. in [7] conducted a study that discusses the construction of single-error-correcting quantum codes for two cases. The first case is a natural decay process of multilevel atoms with a natural kind of decay process, and the second case is a bosonic system which is a qudit amplitude damping channel considered to be obtained by truncating the Fock basis of bosonic modes to the maximum occupation number *q*_−1_ for a single bosonic mode [8]. To correct single amplitude damping (AD), the error correction which is sufficient to detect one *A*_0_ error and to correct *A*_*ij*_ error with *j* = *i* + 1 was used. On the contrary, the cascade structure has been considered for the decay process of multilevel atoms.

A study conducted by Laflamme et al. in [3] proposed a quantum error correction code that protects a qubit against one-qubit errors. A simple circuit is constructed to encode the original state by distributing quantum information over the minimal number required which is five qubits in this study. The circuit can be used for decoding by simply running it backward. By assuming that the interaction affected at most one bit in any way, they had shown that there is a 5-qubit code that has perfect accuracy or fidelity [9]. This paper stated that if the probability of an error in one qubit is *p*, the fidelity of the code where the restriction to only one error is lifted will be 1 − *cp*_2_+⋯, for some constants.

During the last few years, bosonic QEC is demonstrated to reach the break-even point, i.e., the lifetime of a logical qubit is enhanced to exceed that of any individual components composing the experimental system [11–13]. Beyond that, universal gate sets and fault-tolerant operations on the bosonic codes are also realized, pushing quantum information processing towards the QEC era.

## 3. Quantum Error-Correcting Code

In quantum computers, error correction is important to transmit the qubit to the receiver smoothly without any quantum interference or error. QEC is used in quantum computing to protect quantum information from errors due to decoherence and other quantum noise. Quantum error correction is essential if one is to achieve fault-tolerant quantum computation that can deal not only with noise on stored quantum information but also with faulty quantum gates, faulty quantum preparation, and faulty measurements [14–16].

However, the quantum states that carry out the transmission are very sensitive to the imperfections of computer hardware and also decoherence caused by the environment and surrounding interactions. The sensitive condition of the quantum state makes the quantum computation so difficult to practice unless there is error correction which is used. In this section, the three-bit code of method for quantum error-correcting codes is discussed [17].

**Figure 1:**
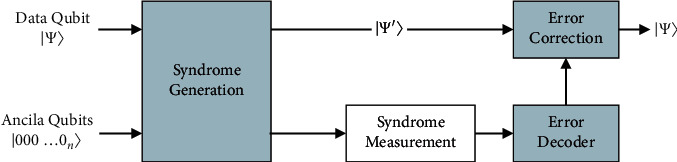
The quantum error correction (QEC) model.

Redundancy is the simplest way of correcting a classical error in which the transmitted information is stored or copied multiple times. This code can be used either to correct a bit error or to detect bit error. It cannot simultaneously do both [18]. To correct the error in the transmitted code word, it follows the majority vote for the optimal algorithm.  Figure 1 shows the quantum error correction (QEC) model.

However, in quantum computers, the copy or redundancy method is not applicable due to the no-cloning theorem. In the no-cloning theorem, it is physically impossible to copy the random unknown quantum state [19]. In a quantum system, the qubit is arranged in the Hilbert space [13–20]. The first quantum error-correcting code was proposed by Peter Shor. Shor has implemented a way of correcting a quantum error by storing quantum information in the entanglement of nine qubits to protect a single qubit. After that, a different scheme is proposed which used only seven bits to correct the quantum error [21].

## 4. Digital System Design for Quantum Error Correction

As known, coding based on the classical and quantum theory of information has many applications on the Internet of Things [22–26]. In quantum computing, three-qubit codes can be used to correct the single-bit flip error and single-phase error at a time. They cannot correct both cases at one time. These codes use entanglement and syndrome measurements to diagnose and correct the error [27].

**Figure 2:**
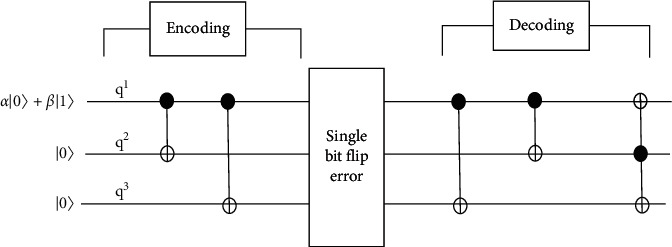
Simple circuit illustrating the principle of quantum error correction.

Suppose that the transmitter wants to transmit a single-qubit state *α|*0+*β|*1 through a noisy communication channel to the receiver. In this case, the noise is assumed to act independently on each qubit and an effect chosen at random for a given qubit between the unchanged state of the qubit and applying a Pauli *σ*_*x*_ operator. The probability for the unchanged state of the qubit is 1 − *p*, and the probability for applying Pauli *σ*_*X*_ operator is *p* < 1/2. This is a very artificial type of noise. However, if it can be corrected, the correction will offer a useful result for other realistic types of noise [28, 29]. This transmission channel will produce *σ*_*x*_ errors randomly. So, the transmitter will add two more qubits of *|*0 into the initial single qubit *α|*0+*β|*1⟶*α|*000+*β|*100.  Figure 2 shows the simple circuit which illustrates the principle of quantum error correction.

Here, the encoder will entangle the two redundant qubits with the input qubit:

1. If the input state is *|*0, then the encoder does nothing, so the output state is *|*000
2. If the input state is *|*1, then the encoder flips the lower states, so the output state is *|*111
3. If the input is a superposition state, then the output is the entangled state *a*|000> + *b*|111>

The decoder looks just like the encoder.

If the input to the decoder is |000> or |111>, there is no error, so the output of the decoder is as follows:

  Input-output
  |000>-|000>
  |111>-|100> (the top 1 causes the bottom bits to flip)

The initial single qubit will experience entanglement with two other qubits of |0) using a controlled NOT (CNOT) gate. The transmitter source operates a CNOT gate from the first qubit (*q*_1_) to the second qubit (*q*_2_) which will produce *α|*000+*β|*110. Then, the first qubit operates the CNOT gate into the third qubit (*q*_3_) and produces *α|*000+*β|*111 which is the final result of the encoded qubit [30, 31]. All these three encoded qubits are sent down into the channel and have been interfered with by noise.

On the decoder side of the circuit, it is assumed that at most 1 qubit will be flipped, and the bit flip is just as likely to affect any qubit. The receiver prepares two more qubits in the state of *|*00. These two qubits are used to diagnose the error in the encoded code [32]. When the encoded code of *α|*000+*β|*111 undergoes decoding process in the CNOT gate, the result will come out as *α|*000+*β|*100. All possible values of the state at the end of the decoder are listed in Table 1. The last two qubits are called the syndrome, and their values indicate the error type that occurred.  Table 2 shows the list of syndromes, errors detected, and how to correct them.

**Figure 3:**
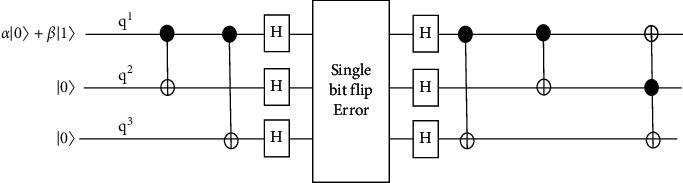
Modified circuit by adding Hadamard gates.

The three-qubit code can also be used to correct single-phase flip in (3,1) circuits. It uses Hadamard (H) gate to convert a sign flip or phase flip *σ*_*Z*_ to bit flip *σ*_*X*_. Phase flip occurs when the sign of a qubit *|*0 and *|*1 becomes inverted. For example, if the initial state of the qubit is *α|*++*β|*− which is corrupted, it will change into *α|*++++*β|*−−−.  Figure 3 shows the circuit that is used to correct the bit flip earlier is modified by adding Hadamard gate [27] at the end of the encoder and the beginning of the decoder to correct the single-phase flip.

If there is no phase flip in the qubit, the Hadamard gate will cancel each other, and if the circuit detected a phase flip, the two Hadamard gates will convert it into a bit flip. The discussion above showed that the three-qubit code can be used to correct a single quantum error in quantum computing without the use of cloning in the encoding. It also showed that this method can correct the errors without damaging the information in the qubit. However, this three-qubit code can only correct bit flips or phase flips at one time. It cannot correct both cases simultaneously.

**Figure 4:**
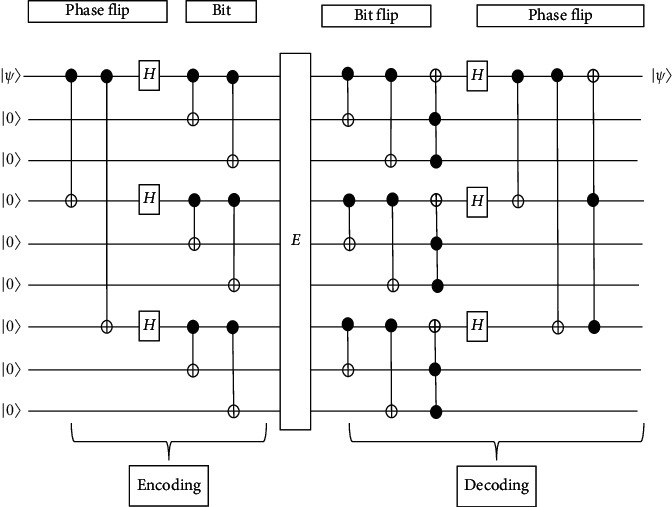
Shor's nine-qubit error-correcting circuit.

The qubit may be corrupted by a bit flip or sign flip or both cases at one time. Shor's nine-qubit error-correcting codes can be used to correct both types of errors. In addition, these codes can also correct arbitrarily corrupted single-qubit (see Figure 4).

First and foremost, the initial qubit state of *α*|0) + *β*|1) will go through the encoding process in the encoding circuit and become a general qubit as

(1) $$\begin{array}{c} \alpha |0\rangle +\beta |1\rangle ⟶\left[\alpha |0\rangle =\frac{1}{\sqrt{2}}\left(|0_{1}0_{2}0_{3}\rangle +|1_{1}1_{2}1_{3}\rangle \right)\left(\frac{1}{\sqrt{2}}\left(|0_{4}0_{5}0_{6}\rangle +|1_{4}1_{5}1_{6}\rangle \right)\right)\left(\frac{1}{\sqrt{2}}\left(|0_{7}0_{8}0_{9}\rangle +|1_{7}1_{8}1_{9}\rangle \right)\right)\right]\\ +\left[\beta \frac{1}{\sqrt{2}}\left(|0_{1}0_{2}0_{3}\rangle -|1_{1}1_{2}1_{3}\rangle \right)\left(\frac{1}{\sqrt{2}}\left(|0_{4}0_{5}0_{6}\rangle -|1_{4}1_{5}1_{6}\rangle \right)\right)\left(\frac{1}{\sqrt{2}}\left(|0_{7}0_{8}0_{9}\rangle -|1_{7}1_{8}1_{9}\rangle \right)\right)\right]. \end{array}$$

**Figure 5:**
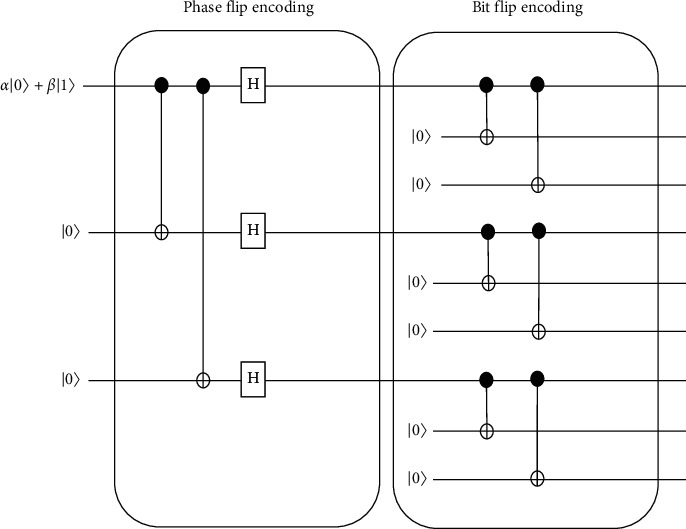
Encoding circuit for nine-qubit codes.

Those nine qubits will undergo phase-flip encoding in the circuit. As shown in Figure 4, the first, fourth, and seventh qubits are fixed for the phase-flip code. Three groups of qubits which are (1,2,3), (4,5,6), and (7,8,9) will be considered as bit-flip groups. If these three groups act as three inputs, the circuit can be reduced as the phase-flip code.  Figure 5 shows the circuit for phase-flip encoding and the bit-flip encoding that is used in nine-qubit codes.

**Figure 6:**
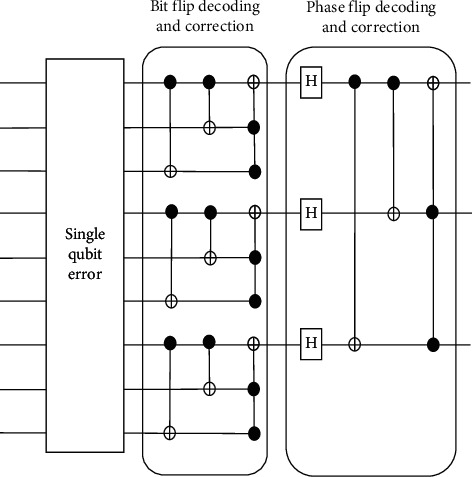
Decoder circuit for nine-qubit codes.

The decoding circuit that is used for the decoding process in the nine-qubit code is shown in Figure 6. After the encoding process, assume that the general qubit from the encoder is corrupted and interrupted with decoherence and other noises and the first qubit has been under the phase-flip and bit-flip error as highlighted in the following:

(2) $$\begin{array}{c} \alpha |0\rangle +\beta |1\rangle ⟶\left[\alpha |0\rangle =\frac{1}{\sqrt{2}}\left(|0_{1}0_{2}0_{3}\rangle -|1_{1}1_{2}1_{3}\rangle \right)\left(\frac{1}{\sqrt{2}}\left(|0_{4}0_{5}0_{6}\rangle +|1_{4}1_{5}1_{6}\rangle \right)\right)\left(\frac{1}{\sqrt{2}}\left(|0_{7}0_{8}0_{9}\rangle +|1_{7}1_{8}1_{9}\rangle \right)\right)\right]\\ +\left[\beta \frac{1}{\sqrt{2}}\left(|0_{1}0_{2}0_{3}\rangle +|1_{1}1_{2}1_{3}\rangle \right)\left(\frac{1}{\sqrt{2}}\left(|0_{4}0_{5}0_{6}\rangle |1_{4}1_{5}1_{6}\rangle \right)\right)\left(\frac{1}{\sqrt{2}}\left(|0_{7}0_{8}0_{9}\rangle -|1_{7}1_{8}1_{9}\rangle \right)\right)\right]. \end{array}$$

The interrupted qubit then goes through the bit-flip decoding and correcting circuit resulting as

(3) $$\begin{array}{c} \alpha |0\rangle +\beta |1\rangle ⟶\left[\alpha |0\rangle =\frac{1}{\sqrt{2}}\left(|0_{1}0_{2}0_{3}\rangle -|1_{1}1_{2}1_{3}\rangle \right)\left(\frac{1}{\sqrt{2}}\left(|0_{4}0_{5}0_{6}\rangle +|1_{4}1_{5}1_{6}\rangle \right)\right)\left(\frac{1}{\sqrt{2}}\left(|0_{7}0_{8}0_{9}\rangle +|1_{7}1_{8}1_{9}\rangle \right)\right)\right]\\ +\left[\beta \frac{1}{\sqrt{2}}\left(|0_{1}0_{2}0_{3}\rangle +|1_{1}1_{2}1_{3}\rangle \right)\left(\frac{1}{\sqrt{2}}\left(|0_{4}0_{5}0_{6}\rangle -|1_{4}1_{5}1_{6}\rangle \right)\right)\left(\frac{1}{\sqrt{2}}\left(|0_{7}0_{8}0_{9}\rangle -|1_{7}1_{8}1_{9}\rangle \right)\right)\right]. \end{array}$$

After that, it will pass through the Hadamard gate in the phase-flip decoder and correcting circuit. After undergoing the correction of bit flip and phase flip, the result will become *α* |0_1_1_4_1_7_) + *β*|1_1_1_4_1_7_>. The first qubit from the result is the protected qubit. So, the final result would be the same as the initial qubit code which is *α* |0> + *β*|1>.

Three cases can be fixed by the nine-qubit error-correcting code which are qubit bit flips, phase flips, and both bit flips and phase flips. To correct the bit flips, the circuit firstly will compare qubits one and two, compare the second qubit and third qubit, and also apply the Pauli *σ*_*x*_ operator when necessary. The same method is used for correcting the phase-flip errors except applying the Pauli *σ*_*z*_ operator instead of *σ*_*x*_. Lastly, for both bit-flip and phase-flip errors, the circuit will independently fix both errors.

## 5. Conclusion

The objective of this paper is to design a digital system for the quantum error correction (QEC) code based on the quantum theory which utilizes the quantum mechanics concept for correcting the errors. Quantum error correction (QEC) is considered as a backbone of quantum information processing since it converts an unmanageable continuum of errors into a manageable discrete set, three-qubit code and Shor's nine-qubit error-correcting code. The paper assumed that the general qubit from the encoder is corrupted and interrupted with decoherence and another noise and the first qubit has been under the phase-flip and bit-flip error. The design is verified and validated for three cases, i.e., qubit bit flips, phase flips, and both bit flips and phase flips, which can be fixed by the nine-qubit error correction code.

## Figures and Tables

**Table 1:** Possible values of the encoder, decoder, and syndrome.

State (encoded)	State (decoded)	Syndrome
*α*|000) + *β*|111)	*α*|000) + *β*|100)	00
*α*|100) + *β*|011)	*α*|111) + *β*|011)	11
*α*|010) + *β*|101)	*α*|010) + *β*|110)	10
*α*|001) + *β*|110)	*α*|001) + *β*|101)	01
*α*|110) + *β*|001)	*α*|101) + *β*|001)	01
*α*|101) + *β*|010)	*α*|110) + *β*|010)	10
*α*|011) + *β*|100)	*α*|011) + *β*|111)	11
*α*|111) + *β*|000)	*α*|100) + *β*|000)	00

**Table 2:** List of syndromes, error detected, and action to correct the error.

Syndrome	Error	Action
00	No error	No action
01	3^rd^ qubit flipped	Apply *σ*_*x*_ to the 3^rd^ qubit
10	2^nd^ qubit flipped	Apply *σ*_*x*_ to the 2^nd^ qubit
11	1^st^ qubit flipped	Apply *σ*_*x*_ to the 1^st^ qubit

## Data Availability

No data were used to support the findings of this study.
